# Supplementary material for: Enhanced Adsorptivity of Hexavalent Chromium in Aqueous Solutions Using CTS@nZVI Modified Wheat Straw-Derived Porous Carbon
Source: Nanomaterials (Basel). 2024 Jun 3;14(11):973. doi: 10.3390/nano14110973 (PMC11173464; doi:10.3390/nano14110973)
Supplement: Supplementary file 1 [file nanomaterials-14-00973-s001.zip › nanomaterials-3013617-supplementary.pdf]

Supplementary Materials

Enhanced Adsorptivity of Hexavalent Chromium in Aqueous Solutions Using CTS@nZVI Modified Wheat Straw-Derived Porous Carbon

Tiantian Deng [1,*], Hansheng Li [1,2], Su Ding [1], Feng Chen [1], Jingbao Fu [1] and Junwei Zhao [3]

[1] School of Environmental and Biological Engineering, Henan University of Engineering,
Zhengzhou 451191, China; hansonlee1989@gmail.com (H.L.); sue_ding189@163.com (S.D.); chenfeng871588@163.com (F.C.); fujingbao@126.com (J.F.)

[2] Faculty of Health Sciences, University of Technology MARA, Puncak Alam Campus,
Puncak Alam 42300, Malaysia

[3] College of Resources and Environment, Yangtze University, Wuhan 434023, China; zjw882@139.com

* Correspondence: tt18100335215@163.com or ttdeng0227@haue.edu.cn; Tel.: +86-037162508218

1. Supplementary Experimental

1.1 Materials

The reagents employed in this study were of analytical grade and included the following: hydrogen nitrate (HNO_3, 98%); hydrochloric acid (HCl, 36%～38%); ortho-phosphoric acid (H_3PO_4, 85%); sulphuric acid (H_2SO_4, 98%); sodium hydroxide (NaOH, 96%); potassium hydroxide (KOH, 95%); iron sulfate heptahydrate ($FeSO_4{\cdot}7H_2O$); sodium borohydride ($NaBH_4$, 99%); absolute ethanol (C_2H_5OH); diphenylcarbazide ($C_{13}H_{14}N_4O$, 98%); solid Potassium dichromate ($K_2Cr_2O_7$, 99.5%).The above drugs were purchased from Maclean's Reagent Company.

1.2 Materials Characterization

The Raman spectrometer (InVia, Renishaw, England) was employed to acquire molecular vibration information and analyze the structural characteristics based on the distinctive peaks of the material. Fourier-transform infrared spectrometry was conducted using a Nicolet 6700 instrument (Thermo Fisher, America). For material morphology analysis and surface characteristic observations, scanning electron microscope(Sigma500, ZEISS, Germany) with an energy dispersive spectrometer (EDS) was utilized. The N_2 adsorption–desorption isotherms were obtained by a specific surface area and porosity analyzer (Autosorb iQ 2 MP-XR, Boynton Beach, USA) at the liquid nitrogen condition. The Brunauer–Emmett–Teller (BET) method and Barrett–Joyner–Halenda (BJH) method were used to calculate the specific surface area and pore size distribution of the samples according to the nitrogen adsorption data, respectively.Phase analysis and determination of the crystal structure characteristics were carried out using an X-ray diffraction instrument (D8 ADVANCE, Bruker, Germany), with comparisons made for accurate characterization. The X-ray photoelectron spectrometer (Thermo Kalpha, America) was used for qualitative and semi-quantitative analysis of the elemental composition, chemical state and molecular structure of the sample surface, and the element content or concentration of the sample surface can be obtained from the peak strength.

2. Supplementary Equation

2.1 Analysis and statistic methods of Cr (VI) and Fe

The concentrations of Cr(VI) and Fe were measured viadiphenylcarbazide (at λ = 540 nm) and phenanthroline (at λ=510 nm) spectroscopic methods, respectively using UV spectrophotometry.

The removal rate η(Eq(S1))and the equilibrium adsorption capacity Qe(Eq(S2)) for Cr，were confirmed to illustrate the adsorption effect. They are calculated by the following formula:

$$\eta = \frac{(C_0 - C_e)}{C_0} \times 100\% \quad \text{Eq(S1)}$$

$$Q_\mathrm{e} = \frac{(C_0 - C_e) \times V}{m} \quad \text{Eq(S1)}$$

Here, C0 and Ce represent the initial concentration of Cr (VI) and the equilibrium concentration level at the end of the reaction, mg/L. Qe represents the equilibrium adsorption of the reaction, mg/ g. V is the reaction volume of the Cr (VI) solution, mL. And m is the mass of the adsorbent added, g.

2.2 Fitting and analysis method

Three common models for isothermal adsorption included Langmuir(Eq(S3))、Freudlich(Eq(S4))and Temkin(Eq(s5)) were chosen to relevant adsorption mechanism and the adsorption performance of the adsorbent on Cr (VI).Their equations were shown as follows.

$$Q_e = \frac{Q_m \cdot K_L \cdot C_e}{1 + K_L \cdot C_e} \quad \text{Eq(S3)}$$

$$Q_\mathrm{e} = K_F {C_e}^{1/n} \quad \text{Eq(S4)}$$

$$Q_\mathrm{e} = (\frac{RT}{b}) \ln(AC_\mathrm{e}) \quad \text{Eq(S5)}$$

Among them, Ce is the adsorbent concentration (mg/L/); Qe is the equilibrium adsorption (mg/g), Qm is the maximum adsorption (mg/g); KL is the adsorption equilibrium constant; KF and n are both Freundlich parameters, which related to the adsorbent type and adsorption temperature; A and B are the two constants of the Temkin equation while T is absolute temperature (K) and R represents general gas constant 8.314×10–3 KJ/ (mol·K)

The kinetic data of the adsorption reaction were simulated and analyzed by the pseudo-first-order model(Eq(S6))、pseudo-second-order model(Eq(S7)) and intra-particle diffusion model(Eq(S8)) as shown.

$$Q_\mathrm{t} = Q_e (1 - e^{(-K_1 t)}) \quad \text{Eq(S6)}$$

$$Q_\mathrm{t} = \frac{K_2 {Q_e}^2 t}{1 + K_2 {Q_e}^t} \quad \text{Eq(S7)}$$

$$Q_\mathrm{t} = K_p t^{1/2} + C \quad \text{Eq(S8)}$$

The same parameters have the same meaning as those mentioned above. K_1(1/min), K_2(g/mg·min) are the rate constants of the pseudo-first and pseudo-second order models, respectively. Kp represents the intra-particle diffusion rate constant, and C can be calculated from the Qt and $t^{1/2}$.

In addition, the calculation of adsorption thermodynamics mainly involves the following parameters: Gibbs free energy (ΔG0), enthalpy (ΔH0), entropy (ΔS0), they can be determined by the following equation:

$$\Delta G = -RT\ln K_D \qquad \text{Eq(S9)}$$

$$\ln K_D = \frac{\Delta S^0}{R} - \frac{\Delta H^0}{RT} \qquad \text{Eq(S10)}$$

Where R is the gas constant (8.314 J/ (mol·K)), T is the absolute temperature (K), and KD can be obtained by data calculation.

3. Supplementary Figure

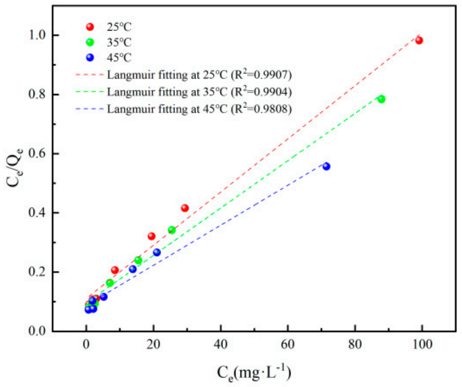

(a)

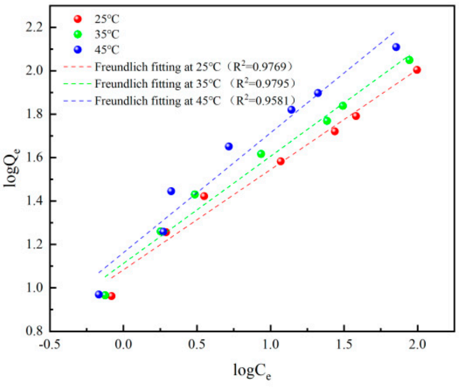

(b)

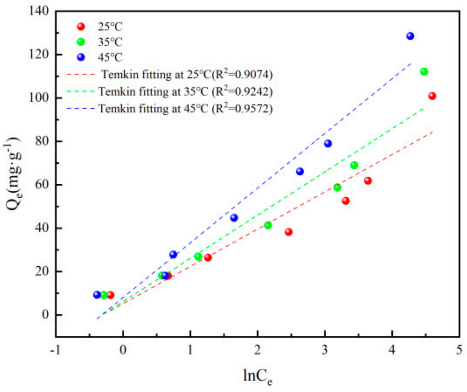

(c)

Figure S1. Adsorption isotherm models with CTS@nZVI-WSPC(a)Langmuir;(b)Freundlich;(c)Temkin (react condition:C_0=50mg/L,T=25 ℃ ,t=24h,dose=0.05g,pH=2)

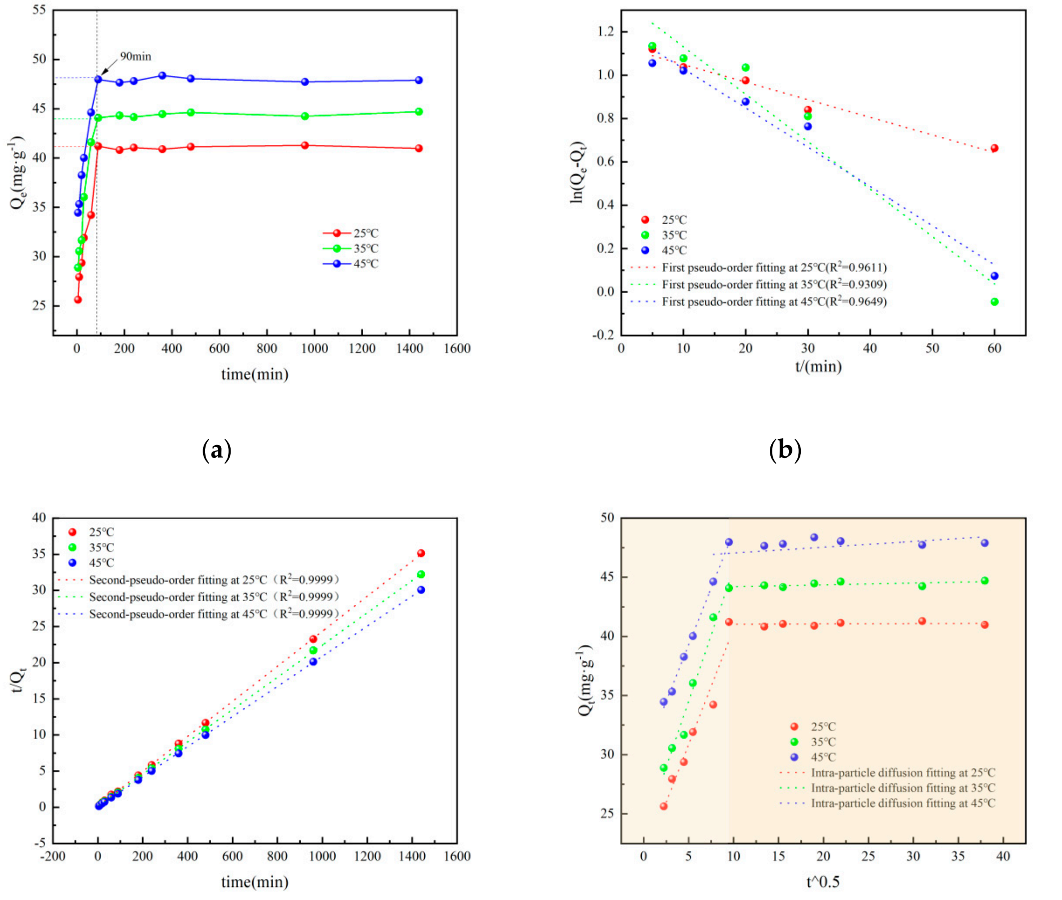

(**a**) (**b**)

(**c**) (**d**)

Figure S2. Effect of time and kinetic models with CTS@nZVI-WSPC(a)Effect of time on adsorptive capacity ;(b) First pseudo fitting;(c)Second pseudo fitting;(d) Intra-particle fitting(react condition :C_0=50mg/L,T=25 ℃ ,t=24h,dose=0.05g,pH=2)

4. Supplementary Table

Table S1. Adsorption thermodynamic parameters

**T (K)**	**ΔG0 (kJ·mol^{-1})**	**ΔH0 (kJ·mol^{-1})**	**ΔS0 (kJ·mol^{-1}·K^{-1})**
**298**	-2.86		
**308**	-3.53	28.31	0.104
**318**	-4.64		
